# Mr. Spry's Case of Poison

**Published:** 1800-12

**Authors:** James Hume Spry

**Affiliations:** Member of the Royal College of Surgeons, London. Aldersgate Street


### *To the Editors of the Medical and Phyſical Journal.*

GENTLEMEN,

IN your Journal for October, a caſe of death from the effects of poiſon appeared, communicated by an ingenious gentleman, Mr. Frederic Thackeray, of Emanuel College, Cambridge. Upon peruſing it, I am induced to ſend you the following caſe and diſſection, which, if you think of ſufficient conſequence to be inſerted in a Journal which very deſervedly ranks high in the eſtimation of medical men, from the candour with which it is conducted, and likewiſe from the many valuable facts with which it abounds, all tending to the promotion of the moſt liberal of all ſciences, the ſcience of medicine, it is much at your ſervice; and if it conveys any information, or if it throws any new light upon the effects of poiſons taken into the alimentary canal, it will prove a ſource of no ſmall ſatisfaction to,

GENTLEMEN,

Your obedient and very humble ſervant,

JAMES HUME SPRY,

Member of the Royal College of Surgeons, London.

*Alderſgate Street, Oct.* 30, 1800.

---

The ſubject of this caſe was an unfortunate young woman, about 27 years of age. I was deſired to viſit her on Monday the 13th of October, about five o'clock in the afternoon; ſhe was reported to be in fits, to which it ſeems ſhe was occaſionally liable.

She was, when I ſaw her, totally inſenſible, agitated by convulſive motions of the whole frame from head to foot; the muſcles of the eyes were much convulſed, and upon elevating the eye-lids, they were rapidly moved in different directions, according as the ſpaſms affected the different muſcles: the pupils were not in a ſtate of dilatation, but rather contracted; light did not appear to have much effect upon them. A viſcid frothy ſaliva oozed from her mouth, which ſeemed ready to obſtruct her breathing; reſpiration was performed with rapidity; ſhe ſighed frequently, and it ſeemed as if there was great oppreſſion about the heart. She moaned conſtantly, as if in great pain; but gave no evidence of ſenſibility, unleſs preſſure was made upon the pit of the ſtomach, when ſhe moved her hand and arm, as if the preſſure gave pain; this part felt unuſually hot to the hand. She was in a very profuſe perſpiration, which had had a very faint earthy ſmell; her pulſe was very ſmall and frequent, but varied occaſionally; her ſtools and urine paſſed involuntarily: theſe ſymptoms gradually increaſed, excepting that the convulſive motions ſubſided, as the comatoſe ſymptoms advanced: The following day, about three o'clock in the afternoon, ſhe breathed her laſt, after having ſuffered the moſt dreadful torments.

From the whole appearance of the patient, which was ghaſtly in the extreme, and from the peculiarity of the different ſymptoms, at the ſame time taking into conſideration the perfect health ſhe enjoyed the preceding day, I had no difficulty in pronouncing, that her death was the conſequence of poiſon: When ſhe took it, or what it was ſhe had taken, was not poſſible to be aſcertained correctly; but from her having been obſerved to be in health on Monday morning, previous to my being called in, which did not happen till the afternoon, it was reaſonable to ſuppoſe, that ſome time during the fore part of that day the fatal potion had been taken.

On Wedneſday, the morning after her deceaſe, in preſence of three medical gentlemen, I examined the body, and the morbid appearances were as follow:—

Upon taking a general view of the body, no external mark of diſeaſe was perceived, if we except that ecchymoſis had taken place in various parts of the back, evidently owing to theſe parts being bruiſed during the convulſive paroxyſms. Putrefaction did not appear to have made much progreſs. Upon opening the thorax, the viſcera contained in it were in general ſound and healthy; the lungs remarkably ſo: the pericardium contained a very ſmall quantity of water: the heart appeared perfectly free from diſeaſe; but upon opening the left ventricle, a polypous connection of coagulable lymph extended through the arterial orifice of the ventricle into the aorta. The œſophagus, as it paſſes down the poſterior part of the thorax, was examined, and was remarkably diſeaſed; it ſeemed to have ſuffered the action of ſome very acrid or cauſtic ſubſtance; the inner membrane, or coat, being in many places deſtroyed; in ſome places it appeared of a whitiſh colour, reſembling the ſlough which is produced by argentum nitratum; in others, gangrenous ſpots were ſituated, and extravaſation of blood had taken place in many other places. Upon opening the abdomen, the viſcera appeared healthy in general; the inteſtines were not particularly diſtended by air; neither was there any particular mark of diſeaſe in the external appearance of any of them, excepting the ileum. The ſtomach, when cut into, ſhewed ſimilar marks of diſeaſe with the œſophagus; near the cardial orifice, there was a ſmall hole* through the villous and nervous coats; adjacent to this hole were a conſiderable number of ſmall red ſpots, which ſeemed to be extravaſations of blood; theſe ſpots occupied a ſpace of about two ſquare inches; the villous coat was eroded in various places; the lower part of the ſtomach was corrugated in a very remarkable manner; (the corrugations were not much unlike the valvulæ conniventes in the ſmall inteſtines) the blood veſſels of this viſcus were not much diſtended; the ſtomach contained about four ounces of a thick viſcid matter, of a dark green colour; the duodenum, jejunum, and ſuperior part of the ileum, were not viſibly diſeaſed; the lower portion of the laſt mentioned inteſtine, for the ſpace of three feet, was very much diſeaſed, both externally and internally; the ſmall veſſels on the ſurface were much diſtended with blood, and appeared as if finely injected. Internally, the villous coat was entirely deſtroyed; the contents of this portion of the ileum were much tinged with blood, from the blood veſſels of the inteſtine being eroded; immediately below that portion of the ileum which was moſt diſeaſed, and between ſeven and eight inches from the valve of the colon, an intus-ſuſceptio was found; and about four inches below it was a ſecond. The effects of the poiſon were not viſible below the ileum; the large inteſtines ſeemed quite in their natural ſtate; the remainder of the abdominal viſcera were perfectly free from any morbid affection whatever: the uterus was unimpregnated.

The contents of the ſtomach and inteſtines were of a ſimilar nature; and I very much lament, that all the experiments which were made to aſcertain the nature of the ſubſtance, or poiſon, which had been ſwallowed, proved abortive.

---

### *To the Editors of the Medical and Phyſical Journal.*

Gentlemen,

I Have read with pleaſure in your uſeful Journal the various proofs which have been adduced in ſupport of the benefits likely to acrue to the community by the introduction of vaccine inoculation.

But, although willing to allow the new practice the merit of

---

* This hole, or ulceration, was ſufficiently large to admit a pea.

